# Expression profiling of ion channel genes predicts clinical outcome in breast cancer

**DOI:** 10.1186/1476-4598-12-106

**Published:** 2013-09-22

**Authors:** Jae-Hong Ko, Eun A Ko, Wanjun Gu, Inja Lim, Hyoweon Bang, Tong Zhou

**Affiliations:** 1 Department of Physiology, College of Medicine, Chung-Ang University, Seoul 156-756, South Korea; 2 Department of Medicine, University of California-San Francisco, San Francisco, CA 94143, USA; 3 Research Center for Learning Sciences, Southeast University, Nanjing, Jiangsu 210096, China; 4 Institute for Personalized Respiratory Medicine, University of Illinois at Chicago, Chicago, IL 60612, USA; 5 Department of Medicine, University of Illinois at Chicago, Chicago, IL 60612, USA

**Keywords:** Ion channel, Breast cancer, Gene expression, p53, Estrogen receptor, Molecular signature, Microarray

## Abstract

**Background:**

Ion channels play a critical role in a wide variety of biological processes, including the development of human cancer. However, the overall impact of ion channels on tumorigenicity in breast cancer remains controversial.

**Methods:**

We conduct microarray meta-analysis on 280 ion channel genes. We identify candidate ion channels that are implicated in breast cancer based on gene expression profiling. We test the relationship between the expression of ion channel genes and p53 mutation status, ER status, and histological tumor grade in the discovery cohort. A molecular signature consisting of ion channel genes (IC30) is identified by Spearman’s rank correlation test conducted between tumor grade and gene expression. A risk scoring system is developed based on IC30. We test the prognostic power of IC30 in the discovery and seven validation cohorts by both Cox proportional hazard regression and log-rank test.

**Results:**

22, 24, and 30 ion channel genes are found to be differentially expressed with a change in p53 mutation status, ER status, and tumor histological grade in the discovery cohort. We assign the 30 tumor grade associated ion channel genes as the IC30 gene signature. We find that IC30 risk score predicts clinical outcome (*P* < 0.05) in the discovery cohort and 6 out of 7 validation cohorts. Multivariate and univariate tests conducted in two validation cohorts indicate that IC30 is a robust prognostic biomarker, which is independent of standard clinical and pathological prognostic factors including patient age, lymph node status, tumor size, tumor grade, estrogen and progesterone receptor status, and p53 mutation status.

**Conclusions:**

We identified a molecular gene signature IC30, which represents a promising diagnostic and prognostic biomarker in breast cancer. Our results indicate that information regarding the expression of ion channels in tumor pathology could provide new targets for therapy in human cancers.

## Background

Ion channels are membrane proteins expressed in various tissues that allow the passage of ions across biological membranes. Ion transport is a key component in a wide variety of biological processes including electrical impulse generation and conduction along nerves, fluid balancing within cells and across cell membranes, and signal transduction within and among cells. In addition, ion channels are known to play critical roles in gene expression, hormone secretion, muscle contraction, immune response, cell volume regulation, and cell proliferation [1-7]. Because of the involvement of ion channels in diverse biological functions, defects in the expression and functional activity of ion channels can cause disease in many tissues [8]. The number of human diseases related with ion channel malfunction has grown rapidly over the past few years [4,9,10]. In particular, there is increasing evidence that ion channels, including both voltage-gated and ligand-gated channels, are involved in the progression and pathology of diversified human cancers [6,7,11-17]. For example, voltage-gated potassium (K^+^) (Kv) channels and calcium (Ca^2+^)-activated K^+^ (K_Ca_) channels are known to control tumor cell proliferation through the modulation of membrane potential in breast, colon, and prostate cancers [12,14,15]. Transient receptor potential (TRP) channels are involved in vascular permeability and angiogenesis and have been implicated in tumor growth and metastasis [18,19]. Several ligand-gated channels, such as nicotinic acetylcholine receptors, affect neoplastic progression by regulating tumor cell proliferation, apoptosis, and angiogenesis [20-23]. More importantly, the expression level of ion channels is potentially able to serve as a prognostic index in human cancers for clinical purposes. For instance, *TRPM1*, a TRP cation channel, is an indicator of melanoma aggressiveness [24] and expression of the Ca^2+^-selective cation channel *TRPV6* is a prognostic marker for tumor progression in human prostate cancer [25]. In addition, the long TRP channel *TRPM8* might serve as a prognostic marker for androgen-unresponsive and metastatic prostate cancer [26] and the expression of *SCN9A*, a voltage-gated sodium (Na^+^) channel, is also useful for prognostic purposes in prostate cancer [27].

Breast cancer is the most common invasive cancer in women worldwide. It is also the principal cause of death from cancer among women globally [28]. A large number of breast cancer studies sought to understand the molecular mechanisms of cancer origin, progression, and invasion that lead to metastasis, and many of these studies have underlined the involvement of ion channels in breast cancer. For example, K_Ca_ channels contribute to breast tumor migration and progression [29,30] and TRP channels are strongly correlated with breast tumor cell proliferation [31]. In addition, the upregulation of several voltage-gated Na^+^ (NaV) channels is associated with metastatic process in breast cancer [32]; however, most of these studies focus on only one ion channel or one type of ion channel. So far, there is no clear picture on the overall expression profiling of different ion channel genes in breast cancer. High-throughput “omic” technologies make it possible to scan all ion channel genes rather than focusing on a single gene or gene family [33]. In this study, we looked to identify molecular signatures consisting of multiple genes from different ion channel families that are implicated in the pathology of human breast cancer. Firstly, we investigated the association of ion channel genes with p53 mutation status in breast tumors. The tumor suppressor p53 is known to play a critical role in regulating the cell cycle and is thus involved in preventing cancer. Mutations in p53 are strongly associated with poor clinical outcome in breast cancer patients [15,23]. Comparison between the p53 mutant and wild-type groups showed that ion channel genes are associated with more aggressive and therapeutically refractory tumors [15]. Secondly, we identified the ion channel genes that were differentially expressed between estrogen receptor (ER) positive and negative breast cancer patients. About 75% of all breast cancers are ER positive, which grow in response to the hormone estrogen. ER is a powerful prognostic marker and an efficient target for the treatment of hormone-dependent breast cancer [26]. Identification of the ER-related ion channels helps us understand the role of ER in the development and progression of breast cancer. Thirdly, we investigated the relationship between ion channel gene expression and histological tumor grade in breast cancer. We identified a molecular signature consisting of 30 ion channel genes (IC30), which significantly correlated with tumor grade. We demonstrate that IC30 is a robust prognostic biomarker to predict clinical outcome in breast cancer, and is independent of standard clinical and pathological prognostic factors including patient age, lymph node status, tumor size, tumor grade, ER status, and progesterone receptor (PR) status. The performance of IC30 was also validated in several independent cohorts from different parts of the world (Table 1).

**Table 1:** Gene expression datasets of breast cancer

**Organization of data source**	**Abbreviation**	**GEO accession**	**Reference**
Genome Institute of Singapore, Singapore	SIN	GSE3494	[15]
Institut Paoli-Calmettes Marseille, France	FRA	GSE21653	[16]
Siemens Medical Solutions Diagnostics GmbH, Germany	GER	GSE11121	[34]
Netherlands Cancer Institute, Netherlands	NED	-^a^	[21]
Karolinska Institutet, Sweden	SWE	GSE1456	[18]
Koo Foundation SYS Cancer Center, Taiwan	TWN	GSE20685	[19]
Nuvera Biosciences Inc, United States	USA1	GSE25066	[20]
Veridex LLC, United States	USA2	GSE2034	[27]

^a^Expression data for NED are publicly available from http://bioinformatics.nki.nl/data.php.

## Results

### Differentially expressed ion channel genes between p53 mutant and wild-type tumors

**Figure 1:**
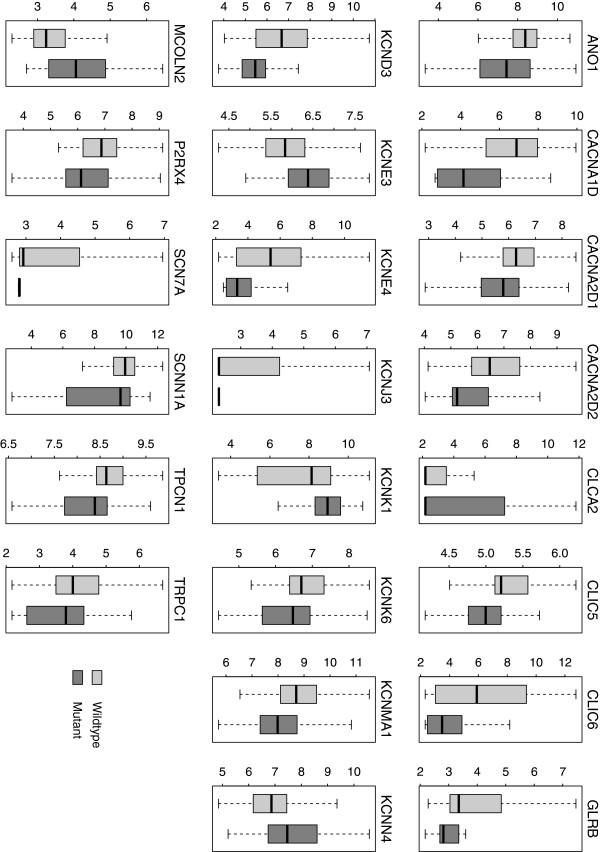
**Boxplot of expression of the genes differentially expressed between p53 mutant and wild-type tumors.** Twenty-two ion channel genes were found to be differentially expressed between the two groups. Light-grey represents wild-type group while dark-grey represents mutant group. Y-axis: log2-transformed expression values.

280 ion channel genes were collected for this study (Additional file 1: Table S1). We aim to identify candidate ion channels that are implicated in breast cancer based on gene expression profiling. We first explored the difference in ion channel gene expression between p53 mutant and wild-type breast tumors in the discovery SIN cohort. There were 58 samples with p53 mutations resulting in protein-level changes and 193 samples with a wild-type genotype [15]. In total, 22 ion channel genes were identified as differentially expressed between the two groups. Five ion channel genes were upregulated in p53 mutant tumors, including *KCNE3*, *KCNN4*, and *MCOLN2*; while 17 ion channel genes were downregulated, including *ANO1*, *KCNMA1*, and *TPCN1* (Table 2 and Figure 1). Among these differentially expressed genes, all the Ca^2+^ channel (*CACNA1D*, *CACNA2D1*, and *CACNA2D2*) and Na^+^ (*SCN7A* and *SCNN1A*) channel genes were downregulated in mutant tumors. In contrast, the expression pattern of K^+^ channel and chloride (Cl^-^) channels was more heterogeneous. Genome-scale inspection indicated a significant enrichment of ion channel genes among the genes regulated by p53 mutation status (*P* = 0.027 by Fisher’s exact test).

**Table 2:** Comparison in gene expression level between p53 mutant and wild-type tumors

**Gene symbol**	**Gene title**	**Fold change**^**a**^	**Adjusted**
			***P*-value**^**b**^
*ANO1*	anoctamin 1, calcium activated chloride channel	0.66	0.001
*CACNA1D*	calcium channel, voltage-dependent, L type, alpha 1D subunit	0.32	< 0.001
*CACNA2D1*	calcium channel, voltage-dependent, alpha 2/delta subunit 1	0.79	0.002
*CACNA2D2*	calcium channel, voltage-dependent, alpha 2/delta subunit 2	0.47	< 0.001
*CLCA2*	chloride channel accessory 2	2.53	0.039
*CLIC5*	chloride intracellular channel 5	0.77	< 0.001
*CLIC6*	chloride intracellular channel 6	0.28	< 0.001
*GLRB*	glycine receptor, beta	0.32	< 0.001
*KCND3*	potassium voltage-gated channel, Shal-related subfamily, member 3	0.46	< 0.001
*KCNE3*	potassium voltage-gated channel, Isk-related family, member 3	1.34	< 0.001
*KCNE4*	potassium voltage-gated channel, Isk-related family, member 4	0.25	< 0.001
*KCNJ3*	potassium inwardly-rectifying channel, subfamily J, member 3	0.51	< 0.001
*KCNK1*	potassium channel, subfamily K, member 1	1.44	< 0.001
*KCNK6*	potassium channel, subfamily K, member 6	0.80	0.010
*KCNMA1*	potassium large conductance calcium-activated channel, subfamily M, alpha member 1	0.65	< 0.001
*KCNN4*	potassium intermediate/small conductance calcium-activated channel, subfamily N, member 4	1.69	< 0.001
*MCOLN2*	mucolipin 2	1.60	< 0.001
*P2RX4*	purinergic receptor P2X, ligand-gated ion channel, 4	0.73	< 0.001
*SCN7A*	sodium channel, voltage-gated, type VII, alpha subunit	0.29	< 0.001
*SCNN1A*	sodium channel, non-voltage-gated 1 alpha subunit	0.71	< 0.001
*TPCN1*	two pore segment channel 1	0.77	< 0.001
*TRPC1*	transient receptor potential cation channel, subfamily C, member 1	0.72	0.005

^a^Fold change is calculated by dividing the mean expression of p53 mutant tumor by the mean expression of p53 wild-type tumor.

^b^*P*-value is calculated by two-tailed *t*-test and adjusted by Benjamini & Hochberg correction.

**Figure 2:**
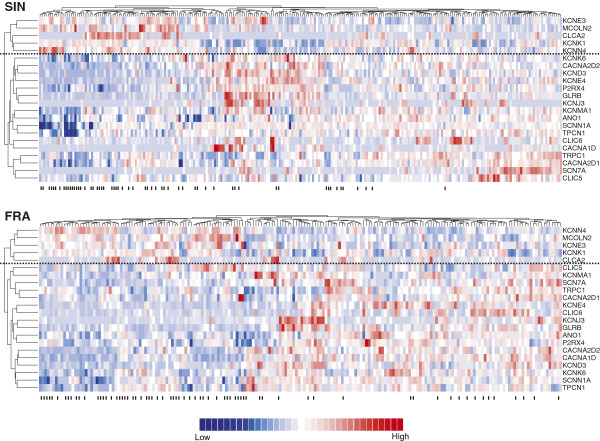
**Heatmaps of expression of the genes differentially expressed between p53 mutant and wild-type tumors.** The differentially expressed ion channel genes were derived from the discovery cohort (SIN) and verified in the FRA cohort. Each row in the heatmaps was labelled with the corresponding gene symbol. The columns labelled with “-” denote p53 mutant tumors. Red represents relatively increased gene expression while blue represents down-regulation. The horizontal dotted line separates the down- and up- regulated gene clusters.

**Figure 3:**
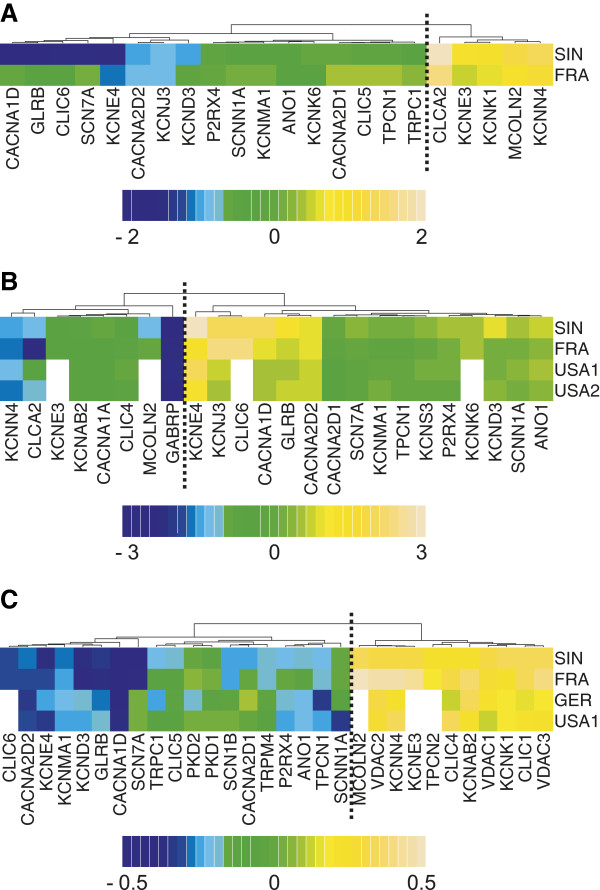
**Validation of ion channel gene expression profiling in breast cancer. (A)** Heatmap of log2-transformed fold change of gene expression level between p53 mutant and wild-type tumors (mutant/wild-type). In the SIN cohort, 5 genes were up-regulated (log2-transformed fold change > 0) while 17 genes were down-regulated (log2-transformed fold change < 0) in mutant tumors. The vertical dotted line separates the down- and up- regulated gene clusters (*AU* = 1 for both clusters). **(B)** Heatmap of log2-transformed fold change of gene expression level between ER positive and negative groups (ER positive/negative). In the SIN cohort, 16 genes were up-regulated (log2-transformed fold change > 0) while 8 genes were down-regulated (log2-transformed fold change < 0) in ER positive patients. The white cells in the heatmap mean the gene expression data are unavailable in the corresponding datasets. The vertical dotted line separates the down- and up- regulated gene clusters (*AU* = 0.995 for both clusters). **(C)** Heatmap of Spearman’s rank correlation coefficient. We found 30 ion channel genes with significant Spearman’s rank correlation between gene expression and histological grade in SIN cohort. Correlation coefficient > 0 means that gene is upregulated in the patients with higher grade while negative correlation coefficient indicates down-regulation in the patients with higher grade. The white cells in the heatmap mean the gene expression data are unavailable in the corresponding datasets. The vertical dotted line separates the down- and up- regulated gene clusters (*AU* = 1 for both clusters).

To test the reliability of the above results in another cohort, we accessed a publicly available microarray dataset on breast cancer (FRA) where p53 mutation status was known. Unsupervised hierarchical cluster analysis demonstrated a very similar expression pattern of the 22 differentially expressed ion channel genes between the SIN and FRA cohorts (Figure 2). We evaluated the statistical significance in hierarchical cluster analysis by approximately unbiased *P*-value (*AU*) (see Methods for details). In the SIN cohort, the hierarchical clusters of upregulated and downregulated genes were highly robust (*AU* = 0.993 for the upregulated cluster and *AU* = 0.963 for the downregulated cluster). Similar results were obtained for the FRA cohort (*AU* = 0.985 for the upregulated cluster and *AU* = 0.990 for the downregulated cluster). Two-tailed t-tests also indicated that 15 out of the 22 genes were significantly (adjusted *P* < 0.05) dysregulated between p53 mutant and wild-type tumors in the FRA cohort (Additional file 1: Table S2). The direction of differential expression in the SIN cohort was reproduced in the FRA cohort (Figure 3A).

### Differentially expressed ion channel genes between ER positive and negative patients

**Figure 4:**
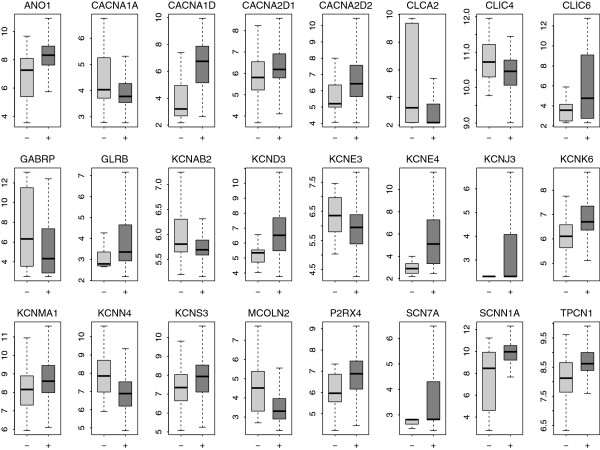
**Boxplot of expression of the genes differentially expressed between ER positive and negative patients.** Twenty-four ion channel genes were found to be differentially expressed between the two groups. Light-grey represents ER negative group while dark-grey represents ER positive group. Y-axis: log2-transformed expression values.

We compared ion channel gene expression between ER positive and negative patients in the SIN cohort. A total of 213 patients were identified as ER positive while 34 patients were identified as ER negative. Twenty-four ion channel genes were identified as differentially expressed between the two groups; 16 genes were upregulated in ER positive patients while 8 genes were downregulated (Table 3 and Figure 4). Among these differentially expressed genes, all the Ca^2+^ channels (except *CACNA1A*) and Na^+^ channel genes were upregulated in the ER positive group, whereas the expression pattern of K^+^ channel and Cl^-^ channels was more heterogeneous. Nineteen out of the 24 differentially expressed genes overlapped with the genes differentially expressed between p53 mutant and wild-type tumors (Figures 1 and 4), which is statistically significant (*P* < 0.001 by cumulative hypergeometric distribution function). Among these common genes, all the downregulated genes in ER positive patients were upregulated in the p53 mutant group and vice versa, which is consistent with previous findings that patients with negative ER status share common pathology with patients harboring mutant p53 [25,35]. We also found a significant enrichment of ion channel genes among the genes dysregulated by ER status (*P* = 0.013 by Fisher’s exact test).

**Table 3:** Comparison in gene expression level between ER positive and negative tumors

**Gene symbol**	**Gene title**	**Fold change**^**a**^	**Adjusted**
			***P*-value**^**b**^
*ANO1*	anoctamin 1, calcium activated chloride channel	2.09	< 0.001
*CACNA1A*	calcium channel, voltage-dependent, P/Q type, alpha 1A subunit	0.65	0.043
*CACNA1D*	calcium channel, voltage-dependent, L type, alpha 1D subunit	5.59	< 0.001
*CACNA2D1*	calcium channel, voltage-dependent, alpha 2/delta subunit 1	1.30	0.033
*CACNA2D2*	calcium channel, voltage-dependent, alpha 2/delta subunit 2	2.61	< 0.001
*CLCA2*	chloride channel accessory 2	0.46	0.017
*CLIC4*	chloride intracellular channel 4	0.75	0.008
*CLIC6*	chloride intracellular channel 6	5.86	< 0.001
*GABRP*	gamma-aminobutyric acid (GABA) A receptor, pi	0.20	0.020
*GLRB*	glycine receptor, beta	3.42	< 0.001
*KCNAB2*	potassium voltage-gated channel, shaker-related subfamily, beta member 2	0.72	0.013
*KCND3*	potassium voltage-gated channel, Shal-related subfamily, member 3	2.36	< 0.001
*KCNE3*	potassium voltage-gated channel, Isk-related family, member 3	0.75	0.003
*KCNE4*	potassium voltage-gated channel, Isk-related family, member 4	12.64	< 0.001
*KCNJ3*	potassium inwardly-rectifying channel, subfamily J, member 3	6.22	< 0.001
*KCNK6*	potassium channel, subfamily K, member 6	1.82	< 0.001
*KCNMA1*	potassium large conductance calcium-activated channel, subfamily M, alpha member 1	1.45	0.035
*KCNN4*	potassium intermediate/small conductance calcium-activated channel, subfamily N, member 4	0.42	< 0.001
*KCNS3*	potassium voltage-gated channel, delayed-rectifier, subfamily S, member 3	1.58	0.028
*MCOLN2*	mucolipin 2	0.46	0.002
*P2RX4*	purinergic receptor P2X, ligand-gated ion channel, 4	1.82	< 0.001
*SCN7A*	sodium channel, voltage-gated, type VII, alpha subunit	1.71	0.009
*SCNN1A*	sodium channel, non-voltage-gated 1 alpha subunit	2.00	< 0.001
*TPCN1*	two pore segment channel 1	1.38	< 0.001

^a^Fold change is calculated by dividing the mean expression of ER positive tumor by the mean expression of ER negative tumor.

^b^*P*-value is calculated by two-tailed *t*-test and adjusted by Benjamini & Hochberg correction.

We next checked the expression profiling the 24 ER status related ion channel genes in three independent cohorts (FRA, USA1, and USA2) where ER status was known. The heatmaps generated by unsupervised hierarchical cluster analysis demonstrated an analogous expression profiling for the 24 ion channel genes among the discovery and validation cohorts (Additional file 2: Figures S1, S2, S3 and S4). A side-by-side comparison between Table 3 and Additional file 1: Table S3 revealed that the 24 genes were significantly (adjusted *P* < 0.05) dysregulated by ER status in at least one out of the three validation cohorts. The direction of differential expression in the SIN cohort was consistent with that in the FRA, USA1, and USA2 cohorts (Figure 3B).

### Correlation between tumor grade and expression of ion channel genes

**Figure 5:**
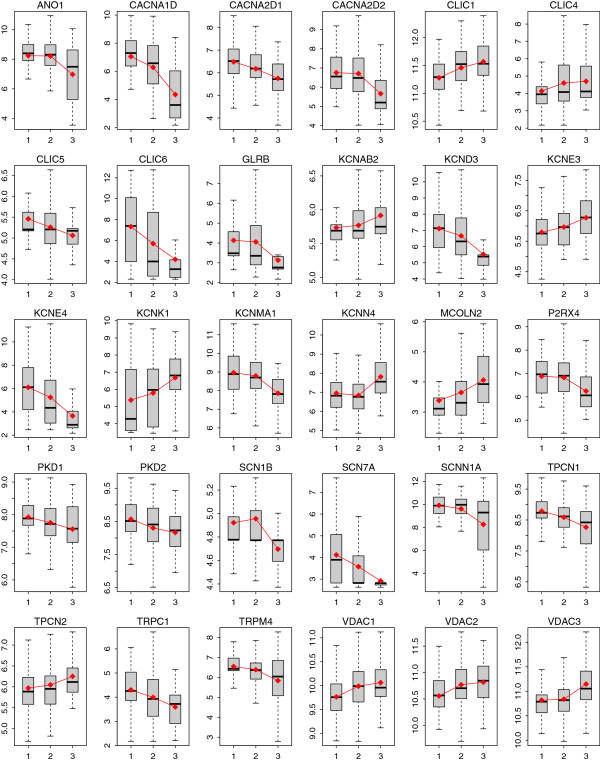
**Boxplot of expression of the 30 ion channel genes associated with histological grade.** The red points and lines indicate the geometric mean of expression in each category. X-axis: histological grade of breast cancer; Y-axis: log2-transformed expression values.

To determine the relationship between tumor progression and ion channel gene expression, we linked gene expression level with histological tumor grade in the SIN cohort, using the Spearman’s rank correlation test. The expression of 30 ion channel genes was found to be significantly (adjusted *P* < 0.05) correlated with tumor grade (Table 4). Eleven out of the 30 genes showed a positive correlation between expression and tumor grade while the other 19 genes showed a negative pattern (Figure 5). Given the fact that tumor grade reflects the differentiation of breast cancer cells, we identified 11 upregulated and 19 downregulated ion channel genes in more aggressive breast tumors. Among these 30 genes, 19 genes were also differentially expressed between the p53 mutant and wild-type tumors. The number of the overlapping dysregulated genes was statistically significant (*P* < 0.001 by cumulative hypergeometric distribution function). Positive correlation between expression and tumor grade corresponds to upregulation in p53 mutant tumors and vice versa, which confirms the well-established findings that p53 mutations link to higher-grade breast cancer and potentially poorer clinical outcomes [32,33,36,37]. Similar to the results described above for p53/ER status, ion channel genes were also significantly enriched among all the genes that were regulated by tumor grade (*P* = 0.003 by Fisher’s exact test).

**Table 4:** Correlation between gene expression and histological tumor grade

**Gene symbol**	**Gene title**	**ρ**^**a**^	**Adjusted**
			***P*-value**^**b**^
*ANO1*	anoctamin 1, calcium activated chloride channel	-0.23	< 0.001
*CACNA1D*	calcium channel, voltage-dependent, L type, alpha 1D subunit	-0.42	< 0.001
*CACNA2D1*	calcium channel, voltage-dependent, alpha 2/delta subunit 1	-0.28	< 0.001
*CACNA2D2*	calcium channel, voltage-dependent, alpha 2/delta subunit 2	-0.30	< 0.001
*CLIC1*	chloride intracellular channel 1	0.26	< 0.001
*CLIC4*	chloride intracellular channel 4	0.16	0.022
*CLIC5*	chloride intracellular channel 5	-0.22	0.001
*CLIC6*	chloride intracellular channel 6	-0.33	< 0.001
*GLRB*	glycine receptor, beta	-0.35	< 0.001
*KCNAB2*	potassium voltage-gated channel, shaker-related subfamily, beta member 2	0.15	0.023
*KCND3*	potassium voltage-gated channel, Shal-related subfamily, member 3	-0.39	< 0.001
*KCNE3*	potassium voltage-gated channel, Isk-related family, member 3	0.23	< 0.001
*KCNE4*	potassium voltage-gated channel, Isk-related family, member 4	-0.38	< 0.001
*KCNK1*	potassium channel, subfamily K, member 1	0.25	< 0.001
*KCNMA1*	potassium large conductance calcium-activated channel, subfamily M, alpha member 1	-0.30	< 0.001
*KCNN4*	potassium intermediate/small conductance calcium-activated channel, subfamily N, member 4	0.23	< 0.001
*MCOLN2*	mucolipin 2	0.25	< 0.001
*P2RX4*	purinergic receptor P2X, ligand-gated ion channel, 4	-0.24	< 0.001
*PKD1*	polycystic kidney disease 1 (autosomal dominant)	-0.17	0.012
*PKD2*	polycystic kidney disease 2 (autosomal dominant)	-0.19	0.004
*SCN1B*	sodium channel, voltage-gated, type I, beta subunit	-0.27	< 0.001
*SCN7A*	sodium channel, voltage-gated, type VII, alpha subunit	-0.41	< 0.001
*SCNN1A*	sodium channel, non-voltage-gated 1 alpha subunit	-0.18	0.008
*TPCN1*	two pore segment channel 1	-0.26	< 0.001
*TPCN2*	two pore segment channel 2	0.17	0.013
*TRPC1*	transient receptor potential cation channel, subfamily C, member 1	-0.25	< 0.001
*TRPM4*	transient receptor potential cation channel, subfamily M, member 4	-0.21	0.002
*VDAC1*	voltage-dependent anion channel 1	0.21	0.002
*VDAC2*	voltage-dependent anion channel 2	0.22	0.001
*VDAC3*	voltage-dependent anion channel 3	0.26	< 0.001

^a^ρ is the Spearman’s rank correlation coefficient.

^b^*P*-value is calculated by Spearman’s rank correlation test and adjusted by Benjamini & Hochberg correction.

Because of the availability of tumor grade information in the FRA, GER, and USA1 cohorts, we tested the expression pattern of the above 30 genes in these 3 independent datasets. We observed a significant correlation between the expression and tumor grade in at least one validation cohort for each gene, except for *TPCN2* (Additional file 1: Table S4). The correlation coefficients for each gene were largely concordant across the discovery and validation cohorts (Figure 3C).

### Prognostic molecular signature composed of ion channel genes

**Figure 6:**
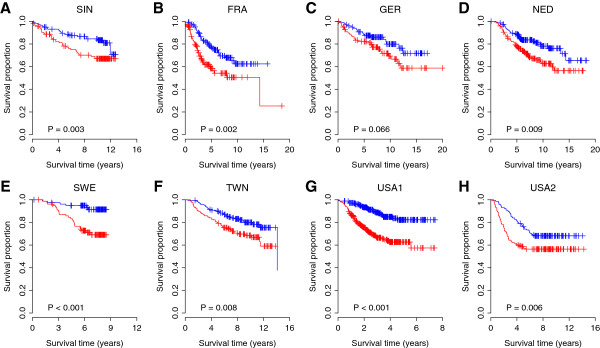
**Kaplan-Meier curves for the patients from eight independent breast cancer cohorts.** Panels from **A** to **H** denote the SIN, FRA, GER, NED, SWE, TWN, USA1, and USA2 cohorts, respectively. The expression of IC30 gene signature predicts poor survival in breast cancer. Red curves are for the IC30 positive patients while blue curves are for the IC30 negative patients. IC30 positive patients were defined as those having a IC30 risk score greater than the group median score. P-values were calculated by log-rank tests for the differences in survival between the IC30 positive and negative groups.

We hypothesized that the 30 ion channel genes associated with tumor histological grade would be predictive of tumor outcome in breast cancer patients. We designated these ion channel genes as the IC30 gene signature. We developed a risk scoring system that combined gene expression information in the IC30 with the Spearman’s rank correlation coefficients (*ρ*) listed in Table 4. IC30 positive patients were defined as those having a risk score greater than the group median score and the other patients were assigned as IC30 negative. We tested the ability of the risk score to stratify patients into prognostic groups in the SIN cohort and the seven validation cohorts (FRA, GER, NED, SWE, TWN, USA1, and USA2). Kaplan-Meier survival analyses were used to compare the IC30 positive and negative groups and determined statistical significance by log-rank tests. The IC30 signature was able to identify patients with poor breast cancer survival in all the cohorts (*P* < 0.01) except for the GER cohort (a marginal *P* = 0.066) (Figure 6). This association between IC30 status and survival was also confirmed by univariate Cox proportional hazard regression of survival. IC30 positive patients had a 1.98-fold increased risk for death in the SIN cohort, 1.99-fold in the FRA cohort, 1.73-fold in the GER cohort, 1.81-fold in the NED cohort, 4.33-fold in the SWE cohort, 1.82-fold in the TWN cohort, 3.11-fold in the USA1 cohort, and 1.71-fold in USA2 cohort (Table 5).

**Table 5:** Univariate Cox proportional hazards regression of survival by IC30 status for patients from 8 cohorts

**Cohort**	**Hazard ratio**	**95% Confidence interval**	***P*-value**
SIN	1.98	(1.15, 3.44)	0.015
FRA	1.99	(1.28, 3.10)	0.002
GER	1.73	(0.96, 3.14)	0.069
NED	1.81	(1.15, 2.86)	0.010
SWE	4.33	(1.76, 10.64)	0.001
TWN	1.82	(1.17, 2.85)	0.008
USA1	3.11	(2.05, 4.70)	< 0.001
USA2	1.71	(1.16, 2.51)	0.006

### Independence of IC30 from other clinicopathological factors

**Figure 7:**
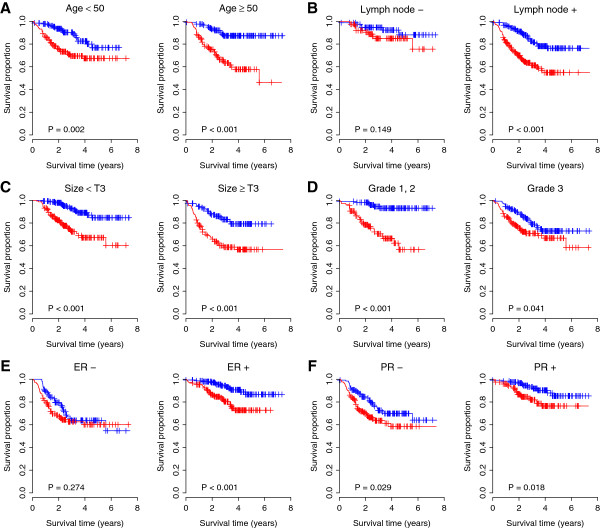
**Kaplan-Meier curves for the patients grouped by clinicopathological factors.** IC30 is independent from other clinicopathological factors in breast cancer. **(A)** Patients were stratified by age; **(B)** Patients were stratified by lymph node status; **(C)** Patients were stratified by tumor size; **(D)** Patients were stratified by tumor grade; **(E)** Patients were stratified by ER status; and **(F)** Patients were stratified by PR status. Red curves are for the IC30 positive patients while blue curves are for the IC30 negative patients. IC30 positive patients were defined as those having a IC30 risk score greater than the group median score. *P*-values were calculated by log-rank tests for the differences in survival between the IC30 positive and negative groups.

We investigated the performance of the IC30 signature in comparison with clinicopathological variables associated with prognosis in breast cancer in the USA1 cohort, the largest dataset in this study. A multivariate Cox regression of survival indicated that IC30 status remained a significant covariate in relation to the standard clinicopathological factors in breast cancer, including patient age, lymph node status, tumor size, tumor grade, and ER and PR status (Table 6). Patients were stratified according to respective clinicopathological factors. For patients aged < 50 and ≥ 50, the IC30 positive patients had a significant 2.37-fold (*P* = 0.003) and 4.21-fold (*P* < 0.001) increased risk for death, respectively. For patients with and without lymph node involvement, the IC30 positive patients had a 2.05-fold (*P* = 0.157) and 2.72-fold (*P* < 0.001) increased risk for death, respectively. For patients with tumor size < T3 and ≥ T3, the IC30 positive patients had a significantly increased risk for death of 3.61-fold (*P* < 0.001) and 2.78-fold (*P* < 0.001), respectively. For patients with lower (1 or 2) and higher (3) tumor grade, the IC30 positive patients had a significantly 6.91-fold (*P* < 0.001) and 1.67-fold (*P* = 0.044) increased risk for death, respectively. For patients with ER negative and positive status, the IC30 positive patients had an increased risk for death of 1.30-fold (*P* = 0.275) and 2.94-fold (*P* = 0.002), respectively. Lastly, IC30 positive patients with PR negative and positive status had a significantly 1.65-fold (*P* = 0.030) and 2.35-fold (*P* = 0.021) increased risk for death, respectively. Kaplan-Meier survival analysis also demonstrated a significantly reduced survival for IC30 positive patients in each subset grouped by age, lymph node status, and tumor size (Figure 7). In addition, univariate Cox regressions of survival confirmed that the IC30 signature was a superior survival predictor in the USA1 cohort, in comparison with age, tumor size, and tumor grade (Additional file 1: Table S5).

**Table 6:** Multivariate Cox proportional hazard regression of survival for the patients from the USA1 cohort

**Covariate**	**Hazard ratio**	**95% Confidence interval**	***P*-value**
IC30 + vs. -	2.21	(1.32, 3.70)	0.002
Age (per year)	1.00	(0.98, 1.02)	0.940
Lymph node + vs. -	2.07	(1.35, 3.16)	< 0.001
Tumor size ≥ T3 vs. < T3	1.73	(1.16, 2.57)	0.007
Grade 3 vs. 1,2	0.67	(0.41, 1.11)	0.119
ER + vs. -	0.58	(0.33, 1.01)	0.055
PR + vs. -	0.76	(0.45, 1.31)	0.330

We also checked the independent predictive power of the IC30 signature in the FRA cohort, where information on age, tumor grade, ER status, PR status, and p53 mutation status is available. A multivariate Cox regression of survival indicated that the IC30 signature was the only significant survival predictor (*P* = 0.014) (Table 7). Univariate Cox regressions of survival also confirmed that the IC30 signature was the most significant prognostic factor in the FRA cohort, in comparison with age, tumor grade, ER and PR status, and p53 mutation status (Additional file 1: Table S6). Taken together, these results suggest that IC30 is associated with clinical outcome and is an independent prognostic factor.

**Table 7:** Multivariate Cox proportional hazard regression of survival for the patients from the FRA cohort

**Covariate**	**Hazard ratio**	**95% Confidence interval**	***P*-value**
IC30 + vs. -	2.55	(1.21, 5.39)	0.014
Age (per year)	1.00	(0.98, 1.02)	0.687
Grade 3 vs. 1,2	0.79	(0.43, 1.45)	0.444
ER + vs. -	0.91	(0.33, 2.52)	0.855
PR + vs. -	1.24	(0.48, 3.18)	0.655
p53 mutant vs. wild-type	1.22	(0.70, 2.12)	0.487

## Discussion

Ion channels are implicated in many physiological processes and also play a pivotal role in the development of cancers; however, it is currently difficult to assign a specific mechanism for each ion channel in the proliferation, invasion, and metastasis of tumor cells [17,24]. Here, we investigated the pathological role of ion channel genes in breast cancer with respect to gene expression level. We tested the association of ion channel genes with p53 mutation status, ER status, and tumor histological grade: 22 ion channel genes were found to be dysregulated between p53 mutant and wild-type tumors, 24 ion channel genes were differentially expressed between ER positive and negative patients, and the expression level of 30 ion channel genes was significantly correlated with histological grade. There is a large overlap between the three differentially expressed gene lists, which suggests a common ion channel-related biological mechanism underlying the different pathological phenotypes. The prognostic value of p53 mutation status has been well characterized in previous studies [15,29-31,35]. The frequency of p53 mutations is higher in ER negative breast cancer [25,35]. Mutant p53 and/or negative ER status are often associated with a high rate of proliferation, a high histological grade, and a poor prognosis [32,33,36,37]. Therefore, it is reasonable that several common ion channel genes are associated with p53 mutation status, ER status, and tumor histological grade, although the causal relationship between these three factors is still controversial.

Gene expression-based molecular signatures have been proven as prognostically valuable in several human cancers [38-42]. Gene signatures that work cooperatively with known clinicopathological factors may enhance prediction accuracy when identifying patients at higher risk for relapse and death. Our proposed molecular signature that is composed of 30 ion channel genes (IC30) associated with tumor grade is a promising prognostic marker. IC30 was solely developed based on the discovery cohort and its prognostic power of IC30 was validated in seven independent validation cohorts. We demonstrated that there were no significant multivariate interactions between IC30 status and other clinicopathological covariates. When grouped by age, tumor size, tumor grade, or PR status, the expression of IC30 further stratified breast cancer patients with significant differences in survival. A significantly increased risk of death was also observed in IC30 positive patients with positive lymph node status or positive ER status. However, a significant difference between IC30 positive and negative groups among the patients with negative ER status was not detected, which may be due to the relatively smaller sample size in this category within the USA1 cohort. In addition, we only detected a marginal significant association for patients with negative lymph node status in the USA1 cohort, and a similar result was reproduced in the GER cohort. In fact, all patients from the GER cohort had negative lymph node status [34] and a marginally significant difference in the risk of death was observed between IC30 positive and negative patients in this cohort. Taken together with previous data, these results confirm that IC30 is not dependent on specific values of the respective covariates status, which enhances the identification of cancer patients at greater risk for death.

Although IC30 gene signature was identified using the tumor grade information, both multivariate and univariate Cox regressions of survival reveal that IC30 is superior to tumor grade, which suggests that the prognostic information contained in IC30 is not limited to tumor grade. Tumor grade only explains a small proportion of variation (less than 25%) in expression of each IC30 gene. The significant association between tumor grade and IC30 gene expression specifically implies that IC30 genes are actively involved in tumor pathology in breast cancer. The combination of 30 genes contains more quantity of information than tumor grade itself. Therefore, IC30 is based on tumor grade but not limited to tumor grade.

The involvement of ion channels in human cancer has been intensively studied in the past years. However, there is no broad consensus on the role and interplay between ion channels and cancer. Generally, ion channels are thought to “assist” cancer by tumor-related cellular behaviors such as proliferation, apoptosis, migration, or angiogenesis [14,17,43,44]. However, it is difficult to assign a detailed role for each ion channel in cancer pathology. In breast cancer, accumulating evidence indicates that K^+^ channels play important roles in regulating tumor cell proliferation, cell cycle progression, and apoptosis [12,45,46]. Although a significant over-expression of K^+^ channels has been correlated with human breast cancer cells [45,47-51], here we report a heterogeneous expression profiling in K^+^ channel genes. Dysregulation in both directions was observed in K^+^ channel genes within the IC30. Four K^+^ channel genes in the IC30 were upregulated in high-grade tumors. For example, *KCNN4*, encoding an intermediate conductance K_Ca_ channel (K_Ca_3.1), is among the upregulated K^+^ channel genes in the IC30 in high-grade tumors. The expression pattern of K_Ca_3.1 was confirmed by a recently published study where K_Ca_3.1 mRNA and protein were more highly expressed in grade 3 tumors than in both grades 1 and 2 [52]. On the contrary, three K^+^ channel genes within the IC30 were downregulated in tumors with higher grade, which includes *KCNMA1* encoding the BK channel alpha subunit. The negative correlation for *KCNMA1* between expression and tumor grade was in accord with four independent cohorts. However, increased expression of *KCNMA1* was found in metastatic breast cancer in the brain compared to metastatic breast cancers in other organs [53], which suggests a more complicated pathological role for the BK channel in tumor metastasis.

Gene expression of Cl^-^ channels also demonstrated a heterogeneous pattern in breast cancer. We reported two up- and two downregulated Cl^-^ intracellular channel genes in high-grade tumors in the IC30. Among them, *CLIC4* was found to be involved in skin cancer [54]; however, the exact role of *CLIC4* is unclear. Besides Cl^-^ intracellular channels, the Ca^+^ activated Cl^-^ channel *CLCA2* was downregulated in breast cancer and is a candidate tumor suppressor gene [55]. We show here that the *CLCA2* gene was upregulated in p53 mutant and/or ER negative breast tumors. In fact, the tumorigenicity of breast cancer was related with a loss of *CLCA2*[56,57]. *CLCA2* is a p53-inducible inhibitor of breast tumor proliferation [55]. However, the reason why *CLCA2* expression is associated with p53 mutation status is beyond the scope of this study.

Ca^2+^ is an essential regulator of the cell cycle and is indispensable for cell proliferation [14]. Increased expression of voltage-gated Ca^2+^ channels has been observed in colon cancer cells [58] and small cell lung cancers [59]. However, all 3 voltage-gated Ca^2+^ ion channel genes in the IC30 were downregulated in p53 mutant tumors and/or high-grade tumors. Apart from these voltage-gated Ca^2+^ channels, the TRP family is also important to provide a Ca^2+^ influx pathway such that Ca^2+^ influx may occur through voltage-gated Ca^2+^ channels [14]. Several studies have demonstrated that the expression of TRP channels is significantly upregulated in breast tumor tissue and breast cancer cell lines [60-63], which is related to malignant growth and cancer progression [64]. However, a paradoxical result was observed in our study. *PKD1*, *PKD2*, and *TRPC1*, which are within the IC30 and belong to the TRP family, were downregulated in patients with p53 mutant tumors and/or of higher histological grade. This expression pattern is consistent in several of the validation cohorts.

Conflicting results were also seen in Na^+^ channel genes. Decreased expression in high-grade tumors was found for the three Na^+^ channel genes in the IC30, which was confirmed by the validation cohorts. However, increased expression of voltage-gated Na^+^ channels has been reported in several cancer types, including breast, prostate, and lung cancer [65-68]. Na^+^ channels were thought to enhance the invasiveness of cancer cells by increasing H^+^ efflux [66] and by stimulating cysteine cathepsin activity [69]. The precise mechanism of Na^+^ channels in tumor development remains unclear [70]. The discrepancy between our results and previous observation may be due to the discrepancy between mRNA expression, protein expression, and channel activity. Our study focused on mRNA abundance. However, protein expression and activity is not directly correlated to mRNA expression. Post-transcriptional regulatory mechanisms predominantly control cellular mRNA to protein abundance ratios [71].

Voltage-dependent anion channels (VDACs) are a class of ion channel located in the outer mitochondrial membrane [72]. We observed a consistent and significant positive correlation between gene expression and tumor grade for the three VDAC genes in the IC30. A recently published study indicated that higher *VDAC1* expression level predicts poor outcome in non-small cell lung cancers [73]. Here, we expanded this finding to breast cancer and the other 2 genes in VDAC family.

## Conclusions

In summary, we investigated the gene expression profile of ion channels in breast cancer with respect to p53 mutation status, ER status, and histological grade. We show that there are numerous common ion channel genes, including *ANO1*, *CACNA1D*, *CACNA2D1*, *CACNA2D2*, *CLIC6*, *GLRB*, *KCND3*, *KCNE4*, *KCNMA1*, *KCNN4*, *MCOLN2*, *P2RX4*, *SCN7A*, *SCNN1A*, and *TPCN1*, that are differentially expressed with a change in p53 mutation status, ER status, and histological grade. The expression pattern of some ion channels, including several potassium, calcium, and sodium channels, is contradictory to previously published results derived from breast cancer cell lines, animal models, and/or human patients. We also identified a molecular gene signature IC30, which represents a promising diagnostic and prognostic biomarker in breast cancer. Further investigation into the role of ion channels in tumor pathology could provide new targets for therapy in multiple human cancers.

## Methods

### Ion channel genes

The definition of human ion channel genes was obtained from IUPHAR-DB [74] and GeneCards [5,9]. In total, we collected 280 ion channel genes, including 5 Ca^+^ activated Cl^-^ channels, 6 Cl^-^ intracellular channels, 9 voltage-sensitive Cl^-^ channels, 1 mid-1-related Cl^-^ channel, 12 K_ca_ channels, 48 Kv channels, 26 voltage-gated Ca^+^ channels, 14 NaV channels, 15 two-pore K^+^ channels, 9 CatSper and two-pore channels, 16 inwardly rectifying K^+^ channels, 4 non-voltage-gated Na^+^ channels, 28 TRP channels, 10 cyclic nucleotide-regulated channels, 20 GABA_A_ receptors, 5 5-HT_3_ receptors, 5 glycine receptors, 18 ionotropic glutamate receptors, 16 nicotinic acetylcholine receptors, 7 P2X receptors, 3 voltage-dependent anion channels, 1 voltage-gated proton channel, 1 voltage-independent cation channel, and 1 zinc activated ligand-gated ion channel (Additional file 1: Table S1).

### Gene expression data

Eight independent microarray breast cancer datasets from Singapore (SIN) [15], France (FRA) [16], Germany (GER) [34], Netherlands (NED) [21], Sweden (SWE) [18], Taiwan (TWN) [19], and the United States (USA1 and USA2) [20,27], were obtained for use in this study (Table 1). These datasets were chosen based on the large number of samples, the availability of clinical outcome data, and the diversity of tumor types. We assigned the SIN dataset as our discovery cohort and the other seven datasets as validation cohorts.

### Microarray data preprocessing

The GC robust multichip average (GCRMA) algorithm [75] in Bioconductor was used to summarize the expression level of each probe set for the microarray data from our discovery cohort (Affymetrix Human Genome U133 set). Only the probe sets present (determined by function “mas5calls” in the Bioconductor “affy” package) in at least one third of the samples were retained. We further limited our analysis to the probe sets with unique annotations and removed genes on chromosomes X and Y to avoid the potential confounding sex factor.

### Statistical analysis

For the SIN and FRA cohorts, a two-tailed *t*-test was used to identify the genes that were differentially expressed between p53 mutant and wild-type tumors. The genes with an adjusted *P*-value < 0.05 after Benjamini & Hochberg correction [22] and fold change > 1.25 were deemed differentially expressed. The same methods and criteria were applied to identify the genes differentially expressed between ER positive and negative patients in SIN, FRA, USA1, and USA2 cohorts.

The Spearman’s rank correlation test was used to detect the relationship between ion channel gene expression level and tumor histological grade. We calculated correlation coefficients and associated *P*-values using the R function “cor.test” with the “spearman” method. The genes with adjusted *P*-value < 0.05 after Benjamini & Hochberg correction were assigned as differentially expressed. We then tested the power of these tumor grade associated genes in predicting clinical outcome in breast cancers. Based on the relationship between gene expression and tumor grade, we can assign a Spearman’s rank correlation coefficient to each gene as a weight (calculated solely from the discovery cohort). A risk score was then calculated for each patient using a linear combination of weighted gene expression as shown below:

$$s=\sum_{i=1}^{n}\rho_{i}\left(e_{i}-\mu_{i}\right)/\tau_{i}$$

Here, *s* is the risk score of the patient; *n* is the number of differentially expressed genes; *ρ*_*i*_ denotes the Spearman’s rank correlation coefficient of gene *i*; *e*_*i*_ denotes the expression level of gene *i*; and *μ*_*i*_ and *τ*_*i*_ are the mean and standard deviation of the gene expression values for gene *i* across all samples, respectively. Patients were then divided into high-score (IC30 positive) and low-score (IC30 negative) groups with the median of the risk score as the threshold value. The median of the risk score was approximately equal to zero in each cohort (Additional file 2: Figure S5). A high score indicated a poor outcome. The “survival” library of the R was used to conduct survival analysis on the risk score.

Hierarchical cluster analysis was conducted to generate the gene expression heatmaps. The statistical significance of hierarchical cluster was evaluated by approximately unbiased *P*-value (*AU*), which is computed by multiscale bootstrap resampling. *AU* of a cluster is a value between 0 and 1, which indicates how strong the cluster is supported by data. Higher *AU* means lower uncertainty of the hierarchical cluster. The “pvclust” library of the R was used to compute the *AU* values.

## Abbreviations

K+: Potassium ion; Ca2+: Calcium ion; Na+: Sodium ion; CL-: Chloride ion; Kv: Voltage-gated potassium channel; KCa: Calcium-activated potassium channel; TRP: Transient receptor potential channel; NaV: Voltage-gated sodium channel; VDAC: Voltage-dependent anion channel; ER: Estrogen receptor; PR: Progesterone receptor; ρ: Spearman’s rank correlation coefficient; AU: Approximately unbiased *P*-value; IC30: Molecular signature consisting of 30 ion channel genes.

## Competing interests

The authors declare that they have no competing interests.

## Authors’ contributions

JHK, EAK, WG, IL, HWB, and TZ designed the study; JHK, EAK, and TZ collected the data. JHK and TZ carried out analyses and prepared figures; HWB conceived of the study; JHK, EAK, WG, IL, HWB, and TZ wrote the manuscript. All authors read and approved the final manuscript.

## Supplementary Material

Additional file 1: Table S1 Ion channel genes involved in this study. **Table S2**. Comparison in gene expression level between p53 mutant and wildtype tumors in validation cohorts. **Table S3.** Comparison in gene expression level between ER positive and negative tumors. **Table S4.** Correlation between gene expression and histological tumor grade. **Table S5.** Comparison in prognostic power between IC30 and clinicopathological factors for the USA1 cohort. Hazard ratio was calculated separately for each variable by univariate Cox proportional hazard regression of survival. **Table S6.** Comparison in prognostic power between IC30 and clinicopathological factors for the FRA cohort. Hazard ratio was calculated separately for each variable by univariate Cox proportional hazard regression of survival. Click here for file

Additional file 2: Figure S1 Heatmaps of expression of the ion channel genes differentially expressed between ER positive and negative tumors. The differentially expressed genes were derived from the discovery cohort (SIN). Each row in the heatmaps was labelled with the corresponding gene symbol. The columns labelled with “-” denote ER positive tumors. Red represents relatively increased gene expression while blue represents down-regulation. **Figure S2.** Heatmaps of gene expression in FRA cohort. The listed genes are differentially expressed between ER positive and negative tumors in the discovery cohort (SIN). Each row in the heatmaps was labelled with the corresponding gene symbol. The columns labelled with “-” denote ER positive tumors. Red represents relatively increased gene expression while blue represents down-regulation. **Figure S3.** Heatmaps of gene expression in GER cohort. The listed genes are differentially expressed between ER positive and negative tumors in the discovery cohort (SIN). Each row in the heatmaps was labelled with the corresponding gene symbol. The columns labelled with “-” denote ER positive tumors. Red represents relatively increased gene expression while blue represents down-regulation. **Figure S4.** Heatmaps of gene expression in USA1 cohort. The listed genes are differentially expressed between ER positive and negative tumors in the discovery cohort (SIN). Each row in the heatmaps was labelled with the corresponding gene symbol. The columns labelled with “-” denote ER positive tumors. Red represents relatively increased gene expression while blue represents down-regulation. **Figure S5.** Distribution of risk score. The red dash lines indicate the median of risk score. There is no significant deviation between zero and the median of risk score in each cohort (|*z*| < 0.2). Click here for file
