# Supplementary material for: Expression profiling of ion channel genes predicts clinical outcome in breast cancer
Source: Mol Cancer. 2013 Sep 22;12:106. doi: 10.1186/1476-4598-12-106 (PMC3849355; doi:10.1186/1476-4598-12-106)
Supplement: Additional file 2: Figure S1 — Heatmaps of expression of the ion channel genes differentially expressed between ER positive and negative tumors. The differentially expressed genes were derived from the discovery cohort (SIN). Each row in the heatmaps was labelled with the corresponding gene symbol. The columns labelled with “-” denote ER positive tumors. Red represents relatively increased gene expression while blue represents down-regulation. Figure S2. Heatmaps of gene expression in FRA cohort. The listed genes are differentially expressed between ER positive and negative tumors in the discovery cohort (SIN). Each row in the heatmaps was labelled with the corresponding gene symbol. The columns labelled with “-” denote ER positive tumors. Red represents relatively increased gene expression while blue represents down-regulation. Figure S3. Heatmaps of gene expression in GER cohort. The listed genes are differentially expressed between ER positive and negative tumors in the discovery cohort (SIN). Each row in the heatmaps was labelled with the corresponding gene symbol. The columns labelled with “-” denote ER positive tumors. Red represents relatively increased gene expression while blue represents down-regulation. Figure S4. Heatmaps of gene expression in USA1 cohort. The listed genes are differentially expressed between ER positive and negative tumors in the discovery cohort (SIN). Each row in the heatmaps was labelled with the corresponding gene symbol. The columns labelled with “-” denote ER positive tumors. Red represents relatively increased gene expression while blue represents down-regulation. Figure S5. Distribution of risk score. The red dash lines indicate the median of risk score. There is no significant deviation between zero and the median of risk score in each cohort (|z| < 0.2). [file 1476-4598-12-106-S2.pdf]

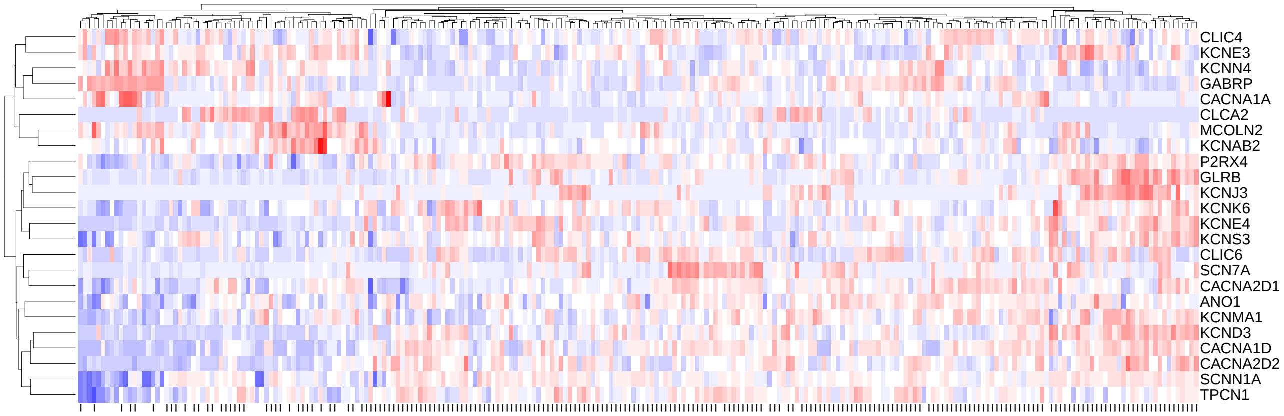

Figure S1: Heatmaps of expression of the ion channel genes differentially expressed between ER positive and negative tumors. The differentially expressed genes were derived from the discovery cohort (SIN). Each row in the heatmaps was labelled with the corresponding gene symbol. The columns labelled with ”-” denote ER positive tumors. Red represents relatively increased gene expression while blue represents down-regulation.

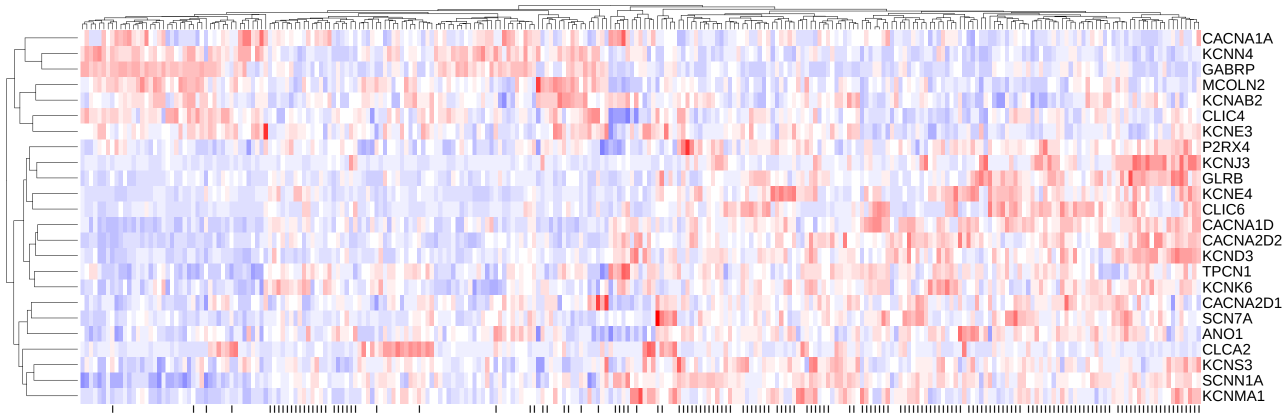

Figure S2: Heatmaps of gene expression in FRA cohort. The listed genes are differentially expressed between ER positive and negative tumors in the discovery cohort (SIN). Each row in the heatmaps was labelled with the corresponding gene symbol. The columns labelled with "-" denote ER positive tumors. Red represents relatively increased gene expression while blue represents down-regulation.

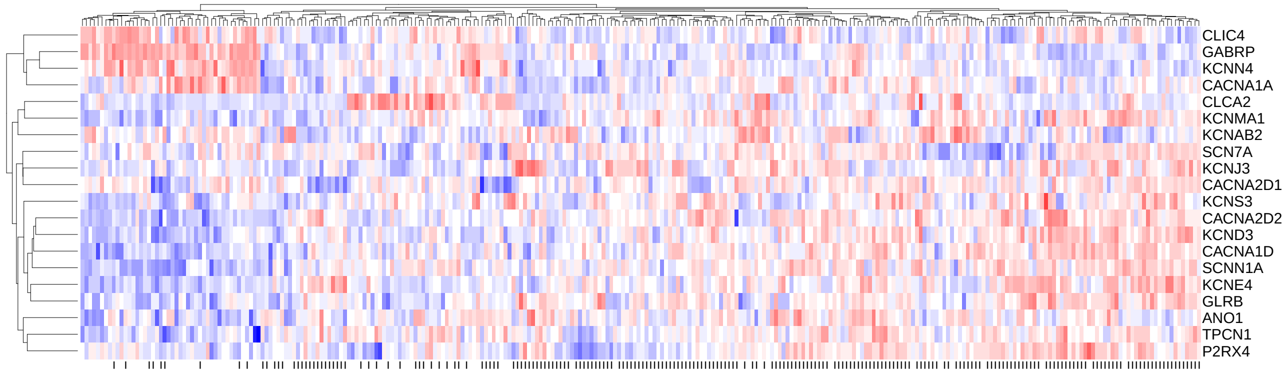

Figure S3: Heatmaps of gene expression in GER cohort. The listed genes are differentially expressed between ER positive and negative tumors in the discovery cohort (SIN). Each row in the heatmaps was labelled with the corresponding gene symbol. The columns labelled with "-" denote ER positive tumors. Red represents relatively increased gene expression while blue represents down-regulation.

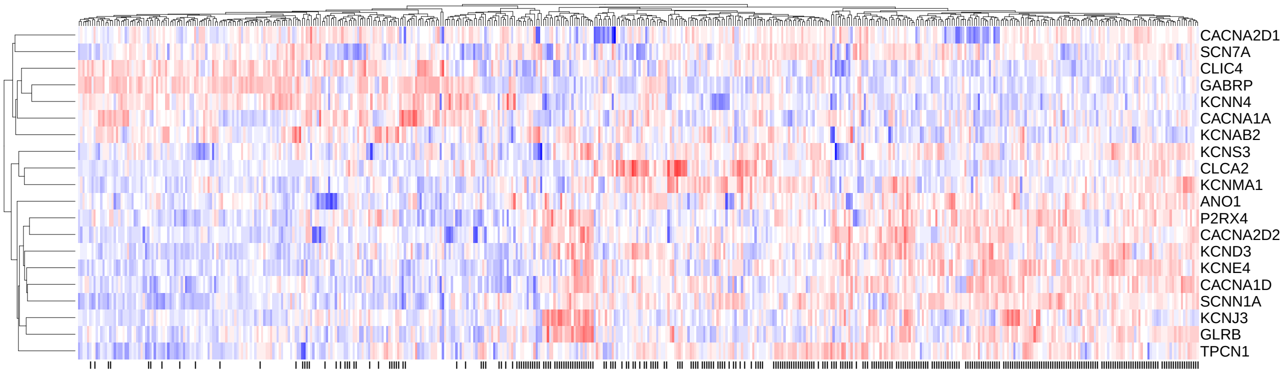

Figure S4: Heatmaps of gene expression in USA1 cohort. The listed genes are differentially expressed between ER positive and negative tumors in the discovery cohort (SIN). Each row in the heatmaps was labelled with the corresponding gene symbol. The columns labelled with "-" denote ER positive tumors. Red represents relatively increased gene expression while blue represents down-regulation.

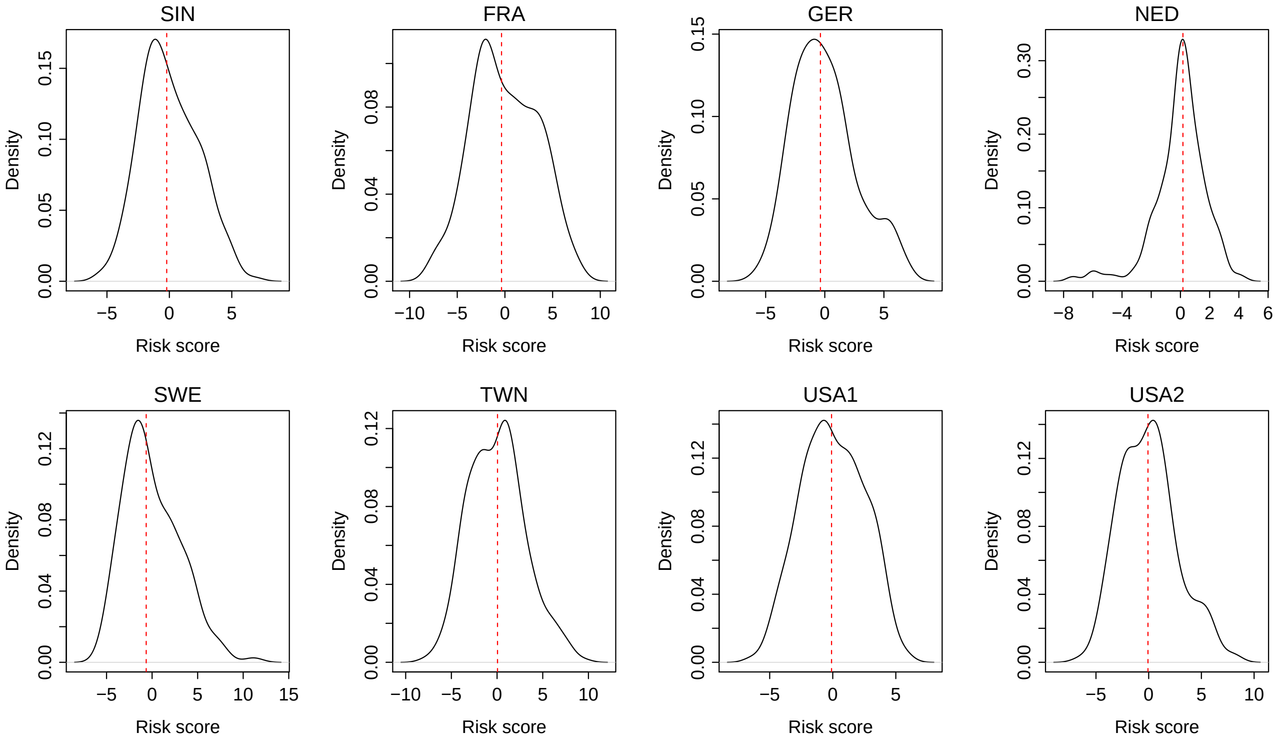

Figure S5: Distribution of risk score. The red dash lines indicate the median of risk score. There is no significant deviation between zero and the median of risk score in each cohort ($|z| < 0.2$).
